# Effect of Packaging and Coating Technique on Postharvest Quality and Shelf Life of *Raphanus sativus* L. and *Hibiscus sabdariffa* L. Microgreens

**DOI:** 10.3390/foods9050653

**Published:** 2020-05-19

**Authors:** Manjula D. Ghoora, Nagarajan Srividya

**Affiliations:** Sri Sathya Sai Institute of Higher Learning (Deemed to be University), Anantapur 515001, Andhra Pradesh, India; manjuladevighoora@sssihl.edu.in

**Keywords:** LDPE bag, PET clamshell, *Aloe vera*, edible coating, pre-harvest spray, dip-coating, postharvest quality, microgreens

## Abstract

Microgreens are highly respiring produce characterized by a relatively short shelf-life. In this study, the efficacy of two types of macro-perforated packaging, PET clamshell (PET–CS) and LDPE self-seal bag (LDPE–SSB), was assessed on the postharvest quality and shelf life of radish (RaS) and roselle (HbS) microgreens stored at 5 °C. Pre-harvest spray treatment (AGSC) was compared with postharvest dip coating (AGDC) using *Aloe vera* gel (AG) for the first time in microgreens for postharvest quality improvement. PET–CS had a lower physiological loss in weight (PLW), respiration rate (RR), electrolyte leakage (EL), microbial counts (MCs), and higher overall acceptability (OA) than LDPE–SSB. AG-coated microgreens had significantly (*p* ≤ 0.05) lesser deteriorative postharvest changes and higher ascorbic acid content than uncoated control. AGSC maintained better OA and postharvest quality than AGDC, especially at the end of the study period in terms of reducing EL, retaining greenness (−a*), and chroma value in HbS microgreens. In RaS microgreens, AGSC helped to maintain lower PLW, MC, and higher ascorbic acid levels. AGSC could be suggested as an eco-friendly ergonomic pre-harvest treatment along with PET–CS for enhancement of postharvest quality and shelf life in RaS and HbS microgreens, with a tremendous potential to be extended to other microgreens.

## 1. Introduction

Microgreens are high-value crops considered the latest innovation in the vegetable sector [1]. Their supply and demand are highly influenced by emerging gastronomic trends and consumer familiarisation with the sensory attributes [2]. However, industrial production and marketing are limited since this highly respiring produce has a very short shelf life at ambient temperature [3,4]. Microgreens are known to respire during the germination process, metabolising stored carbohydrates in the cotyledonary leaves [5]. Once the carbohydrate sources are depleted, degradation of the microgreens occurs. Thus, modification of the plant metabolic activity and extending their shelf life by even a few days could be advantageous.

The main objectives of any postharvest technology are quality optimisation and loss reduction in fresh produce. Modification of the package atmosphere is one of the important methods in extending the shelf life. Passive modified atmosphere packaging (MAP) with relatively high oxygen transmission rate (OTR) films or perforated packaging is suggested to favour postharvest performance in microgreens [2]. High OTR polyethylene bags were preferred for the storage of radish microgreens over laser microperforated polypropylene packaging [6]. In another study, better postharvest quality of “Tah Tasai” Chinese cabbage microgreens was maintained in polyethylene bags compared to polypropylene packaging [3]. Sunflower microgreens have been reported to have a better shelf life and nutritional quality when packed in polystyrene trays compared to LDPE bags [7]. However, at the commercial front, microgreens are mostly packaged in plastic clamshell containers. To the best of our knowledge, there are no scientific reports comparing the efficacy of such commercial packaging with polyethylene packaging on the postharvest quality and shelf life of microgreens. Therefore, such comparison warrants further studies.

Macro-perforated packaging, commonly preferred in commercial set-ups, is known to provide additional gaseous diffusion and is beneficial in reducing off-flavour of fresh produce [8,9]. In addition, our earlier observations have shown that it minimises surface condensation on the packaging used for highly respiring produce, such as microgreens. However, it is often accompanied by higher weight loss, and the content is potentially exposed to outside contaminants. These limitations could be addressed using natural polymeric coating materials as primary packaging on the surface of fresh produce.

Edible coatings represent new packaging strategies in the postharvest management of fresh produce. They are reported to create a micro-modified atmosphere around the produce by acting as a gas and water vapour barrier [10]. This helps in retarding food deterioration and enhancing its quality. Edible coatings are made up of natural polymers such as carbohydrates, proteins, and lipids. Edible coating applications have previously been reported to improve postharvest quality of fresh-cut produce such as celery sticks [11], and minimally processed lettuce [12,13]. However, to date, there are no published reports on the use of edible coating on microgreens.

In the last decade, there has been increased interest in using *Aloe vera* gel, as an edible coating on fruits and vegetables, due to its film-forming, antimicrobial, biodegradable and biochemical properties [14]. Benítez et al. [15] reported *Aloe vera* gel to be a better coating compared to chitosan and alginate coatings to extend the postharvest quality and shelf life of kiwi slices. *Aloe vera* gel (50%) was reported to reduce enzymatic browning in fresh-cut lotus roots and conserve the overall quality when stored at 5 °C [16].

The successful application of the edible coating on foods is dependent on several factors, including the method of application and its cost [17]. Dip coating technique is one of the age-old methods used commercially to coat fresh, whole, and minimally processed fruits and vegetables [18]. In earlier studies conducted in our laboratory, *Aloe vera* gel dip-coating gave promising results by reducing weight loss, minimising changes in the physicochemical parameters, reducing decay and extending the shelf life of papaya [19], figs [20] and litchi fruits [21]. A similar but less pronounced effect was observed in fenugreek and sunflower microgreens (unpublished data) using Aloe gel dip-coating. Dip-coating was found to be a little harsh on delicate and tender microgreens. There are also other drawbacks of dip-coating, such as the requirement of a large quantity of dip solution and quality deterioration of dip solution. Powder-coating was successfully used in our lab instead of dip coating for carrot shreds [22] and radish shreds [23]. However, this is not suitable for microgreens.

The spray-coating technique, which has recently attracted considerable industrial interest [24], was hence considered as an alternative technique. Chitosan postharvest dip-coating alone or combined with preharvest chitosan spray has been reported to enhance fruit quality and lower decay incidence in table grapes [25]. Recently, pre-harvest CaCl_2_ spray has been used to delay senescence in broccoli microgreens [26,27]. In another study, preharvest calcium spray displayed better overall quality and longer shelf life in broccoli microgreens than postharvest dip treatment [28]. However, to the best of our knowledge, there are no published scientific studies evaluating the efficacy of preharvest spray treatment using a bio-based coating such as *Aloe vera* gel on fresh-cut leafy produce or microgreens.

In the present study, radish and roselle microgreens belonging to the Brassicaceae and Malvaceae families, respectively, with different leaf morphologies, were selected. Radish microgreens are characterised by succulent cotyledonary leaves, while roselle have broad, thin, and flat leaves. Based on our nutritional evaluation studies among ten microgreens, these two microgreens were also found to be among the nutrient-rich ones [29], hence the need to optimise their postharvest quality. In the first phase of this study, the postharvest quality of these microgreens was assessed in two macro-perforated packaging, PET clamshell containers, and LDPE self-seal bags, commonly used for packaging fresh-cut produce, salad mixes and microgreens at the commercial and household levels. In the second phase of the study, spray- and dip-coating techniques were compared to study the effect of *Aloe vera* gel as an eco-friendly treatment on the postharvest quality and shelf life of radish and roselle microgreens.

## 2. Materials and Methods

### 2.1. Plant Material Cultivation

Good quality seeds (germination rate > 90%) of radish (RaS; *Raphanus sativus* L.) and roselle (HbS; *Hibiscus sabdariffa* L.) were purchased from government-approved outlets of seed corporations (Anantapur, India). Seeds were broadcast in plastic trays (L: 24 × W: 17 × D: 4 cm) containing cocopeat in triplicates. The seeded trays were germinated in darkness at a relative humidity of 95% ± 5%. After two to three days of germination, they were exposed to sunlight (photoperiod 11.5 h; light intensity 2500–4400 lux) with an average air temperature of 25 ± 5 °C and relative humidity of 65% ± 10%. Seven-day old RaS and HbS microgreens were harvested by cutting the stem ends with sharp and sterile scissors. Microgreens were inspected prior to storage, and plants with defects or discoloured leaves were discarded.

### 2.2. Experimental Design

**Figure 1:**
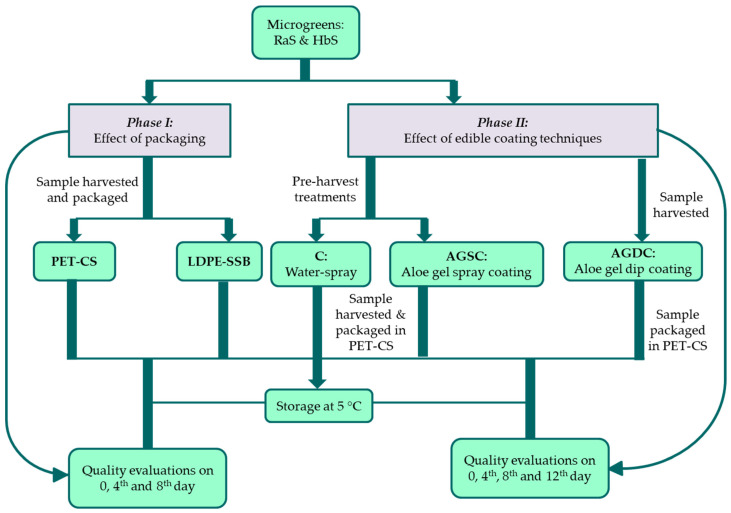
Experimental design and sample coding. RaS: radish microgreens; HbS: roselle microgreens; PET–CS: PET clamshell container; LDPE–SSB: LDPE self-seal bag.

The study was conducted in two phases—Phase I determined the effect of packaging, and Phase II determined the effect of edible coating techniques on the postharvest quality and shelf life of RaS and HbS microgreens in the packaging which maintained better postharvest quality. The summary of the experimental design is represented in Figure 1.

#### 2.2.1. Postharvest Packaging—Phase I

Fifteen grams of freshly harvested microgreens were packaged in clear macro-perforated (4 perforations of 3 mm diameter) polyethylene terephthalate clamshell containers (PET–CS) with hinged lid (dimension: 12.5 × 10 × 3.5 cm; thickness: 0.2 mm) or low-density polyethylene self-seal bags (LDPE–SSB) (dimension: 12.5 × 12.5 cm; thickness: 0.14 mm). The number of perforations was optimised in earlier experiments in our laboratory in order to minimise condensation on the inner package surface and, at the same time, retain the fresh weight of the produce (unpublished data). Samples were stored at 5 °C for 8 days. Quality evaluations were performed on 0, 4 and 8 days of storage, except for physiological loss in weight (PLW), which was measured every two days. Three replicates of each packaging were prepared for every analysis. A total of 84 packaged samples were obtained in Phase I (two microgreens × two packaging × seven parameters × three replicates).

#### 2.2.2. Edible Coating Techniques and Application—Phase II

Medium-sized and freshly harvested *Aloe vera* leaves were used to extract the gel according to a previously standardised protocol [20], and suitable dilutions were prepared for application on RaS and HbS microgreens. Two common techniques viz. spray coating and dip coating, were adopted in this study. The uncoated control (C) comprised of microgreens sprayed with water, prior to harvest. Preliminary trials were conducted to optimise the concentration of *Aloe vera* gel for application (unpublished data). The edible coating comprised of Aloe gel in an amount ranging from about 25 to 50 wt %, with the Aloe gel dip-coating (AGDC) having double the concentration of Aloe gel spray coating (AGSC). Prior to the harvest of microgreens, three trays were randomly selected for the spraying of Aloe gel. The AGSC was applied as a fine mist in the early hours of the morning as multilayers, with intermittent drying periods between the coating application. Harvesting was done upon complete drying of the Aloe gel coating on the surface of microgreens, and they were packaged in PET–CS. In the AGDC treatment, the microgreens were harvested from 3 random trays and dipped in Aloe gel and fan-dried for 5–10 min without allowing wilting to take place. Quality evaluations were performed on 0, 4, 8 and 12 days of storage, except for PLW, which was measured every two days. Three replicates of each treatment were selected for quality evaluations on every sampling day. The final number of packaged samples in Phase II was 126 (two microgreens × three treatments × seven parameters × three replicates).

### 2.3. Quality Evaluations

#### 2.3.1. Physiological Loss in Weight

The physiological loss in weight (PLW) was determined by accurately weighing the bagged samples at the beginning of storage and during storage at regular intervals (every two days). Results were expressed as a percentage of weight loss relative to the initial fresh weight of the microgreens [6].

#### 2.3.2. Respiration Rate

Respiration rates of the microgreens were determined in a closed system every 4 days during the storage period. Preliminary trials were conducted to determine the optimal incubation time for the studied microgreens. Gas samples were taken from the microgreens container every 15 min for a period of 1 h and evaluated until the CO_2_ level reaches a steady point. Thirty minutes was found to be the time when equilibrium was reached. Hence, 30 min was selected as the incubation time. In the case of LDPE–SSB, the macro-perforated package was placed inside a rigid container of a known volume containing ambient air as the initial atmosphere and incubated for 30 mins at 5 °C. This was carried out to minimise handling of the greens and ensure an air-tight atmosphere for gas sampling. In PET–CS containers, the macro-perforations were sealed during the incubation period. Gas composition (O_2_ and CO_2_%) in the headspace of the packaged sample was measured using a needle connected to the CO_2_/O_2_ gas analyser (PBI Dansensor, Checkmate II, Ringsted Denmark). The needle was inserted through a septum (silicone sealant) placed on the rigid container (in the case of LDPE–SSB package) and directly through the septum of the PET–CS containers. The change in the concentration of CO_2_ evolved during the incubation period was used in the calculation of respiration rate using the following Equation (1) [30]:

(1) $$Respiration\;rate\;\left(µL\;CO_{2\;}g^{-1}h^{-1}\;\right)=\frac{\;{\left(\%\;CO_{2}\right)}_{Final}-\;\;{\left(\%\;CO_{2}\right)}_{initial}}{Sample\;weight\;\times \;incubation\;time\;\times 100}\;\times \;headspace\;volume$$

where (% CO_2_) _final_ is the CO_2_ concentration after 30 min; (% CO_2_) _initial_ is the CO_2_ concentration at the beginning of the incubation period; headspace volume is the volume of the container minus the volume occupied by the microgreens, expressed in µL; sample weight is the weight of microgreens on the evaluation day in g; incubation time is expressed in h.

#### 2.3.3. Electrolyte Leakage

Tissue electrolyte leakage was measured following the procedure given by Xiao et al. [31]. Samples (5 g) were submerged in 150 mL deionized water at 20 °C and shaken for 30 min. The electrolyte of the solution was measured using a conductivity meter (ELICO CM-180, India). Total electrolytes were obtained after freezing the samples at −20 °C for 24 h and subsequent thawing. Tissue electrolyte leakage was expressed as a percentage of the total electrolyte.

#### 2.3.4. Instrumental Colour

The instrumental colour of samples was measured with a Konica Minolta colour reader CR-10 (Minolta Co. Ltd., Osaka, Japan), equipped with an 8 mm aperture and calibrated with a white tile before the measurement was performed. The instrumental colour was measured in the form of CIELAB colour coordinates. The colour coordinate L*, which denotes lightness, was measured in both microgreens. To trace the degradation of chlorophyll in the microgreens, a* (−) corresponding to greenness was recorded. The coordinate b* (+) denoting yellowness was measured in RaS as leaf yellowing was observed. In the case of HbS microgreens, since browning and not yellowing was a problem, chroma value, which denotes the overall chromacity, was calculated using the formula (a^2^ + b^2^)^1/2^. Leaves were plucked and placed in a 3-inch petri plate until filled with the sample. The probe of the colour reader was placed onto the adaxial surface of the leaves in the dish, and the reflectance spectra were measured by the instrument directly at three different locations and the mean was calculated.

#### 2.3.5. Ascorbic Acid

The extraction and estimation of free, dehydro- and total ascorbic acid were performed according to the method given by Kampfenkel et al. [32], and the DHA/FAA ratio was computed and expressed. The concentration of ascorbic acid was calculated based on values obtained from the L-ascorbic acid standard curve (100–500 µg/mL). Results were expressed as mg/100 g fresh weight.

#### 2.3.6. Microbial Enumeration

To assess the microbial quality of microgreens, total aerobic mesophilic bacterial count (APC), and total yeast and mold count (YMC) were determined. Aseptically weighed sample (1 g) was homogenised in a sterilised diluent (0.1% peptone water). The extract was centrifuged, filtered under sterile conditions, and volume was made up to 10 mL. The filtrate was serially diluted (10^−1^ to 10^−5^), and 100 µL of the appropriate dilution was spread on the agar plate using a spiral plater. The APC was determined by plating samples on the plate count agar, while YMC was determined by culturing on the potato dextrose agar. The incubation time was 24 and 48 h for APC and YMC, respectively. Microbial colonies were counted using a digital colony counter (Scan100 Interscience, St Nom, France), and results were reported as log CFU/g of sample.

#### 2.3.7. Overall Acceptability and Marketability

Microgreens were evaluated for overall acceptability by a group of 25 female panel members (selected from the authors’ department). The panel members were familiarised with the samples and scoring system, but not specifically trained as they were to reflect consumer acceptability. Samples were coded and presented to the panelists immediately after opening the containers, in a randomised manner. The panelists were asked to rate the samples based on their degree of liking, using a 9-point hedonic scale.

End of shelf life was determined based on marketability score derived from the percentage loss of saleability (Equations (2)–(6); Table 1). The latter was a composite value calculated as a sum of 40% of the degree of wilting, 40% of the degree of yellowing/browning and 20% loss of overall acceptability. The degree of wilting/discoloration was determined by counting the number of wilted/discoloured leaves and expressed as a percentage of the total number of leaves in the package. The parameters were determined on duplicate samples. The overall acceptability (OA), as determined by the sensory panel on a 9-point hedonic scale, was first converted to a percentage. Hundred minus the % OA was the percent loss of acceptability. The equations used to derive the loss of overall saleability are given below:

(2) $$Degree\;of\;wilting\;\left(\%\right)=\frac{Number\;of\;leaves\;wilted}{Total\;number\;of\;leaves\;in\;package}$$

(3) $$Degree\;of\;discolouration\;\left(\%\right)=\frac{Number\;of\;discoloured\;leaves}{Total\;number\;of\;leaves\;in\;package}$$

(4) $$Overall\;acceptability\;\left(\%\right)=⎣\frac{OA\;score}{9}⎦\times 100$$

(5) Loss of overall acceptability (%)=100−% OA

(6) Loss of overall saleability (%)=40% wilting+40% discolouration+20% loss of OA

Marketability scores were assigned as follows:

A loss of saleability of 20–29%, corresponding to a marketability score of ≤3, denoted loss of marketable shelf life of the produce. 

#### 2.3.8. Scanning Electron Microscopy

The surface morphologies of coated (AGSC and AGDC) and uncoated (C) HbS microgreens were observed using an environmental scanning electron microscope (ESEM/VP–SEM–JEOL IT-300, Tokyo, Japan) operated in high vacuum mode. Samples were mounted on an aluminium stub using a double-sided adhesive carbon tape, sputter-coated with a thin layer of platinum and observed.

#### 2.3.9. Statistical Analysis

Three replications per treatment were employed, and results were expressed as means along with their standard deviation. All statistical analyses were performed using SPSS software (IBM SPSS Statistics 25, New York, NY, USA). Data obtained for the seven parameters (PLW, RR, EL, colour, ascorbic acid, microbial quality, and sensory acceptability) across the storage period were subjected to analysis of variance (ANOVA) using the generalised linear model. This was followed by a posthoc Tukey HSD test at *p* ≤ 0.05 to determine significantly different groups. Graphical representations were performed using OriginPro^®^ 2020 Graphing and Analysis software(OriginLab, Northampton, MA, USA).

## 3. Results

### 3.1. Effect of Packaging on Postharvest Quality and Shelf Life of Radish (RaS) and Roselle (HbS) Microgreens

#### 3.1.1. Physiological Loss in Weight (PLW)

**Figure 2:**
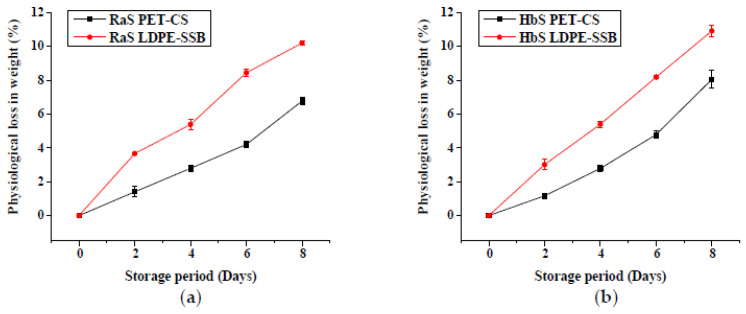
Effect of packaging on the physiological loss in weight in (**a**) radish microgreens (RaS) and (**b**) roselle microgreens (HbS) during storage at 5 °C. PET–CS: PET clamshell containers; LDPE–SSB: LDPE self-seal bags; data are means ± SD (*n* = 3).

Significantly lower PLW (*p* < 0.05) was recorded in samples (Figure 2) stored in PET–CS than LDPE–SSB throughout storage. At the end of 8-day storage, a PLW of 6.8% and 8.1% in PET–CS stored samples, and 10.2% and 10.9% in LDPE–SSB stored samples were recorded in RaS and HbS microgreens, respectively.

#### 3.1.2. Respiration Rate (RR)

**Figure 3:**
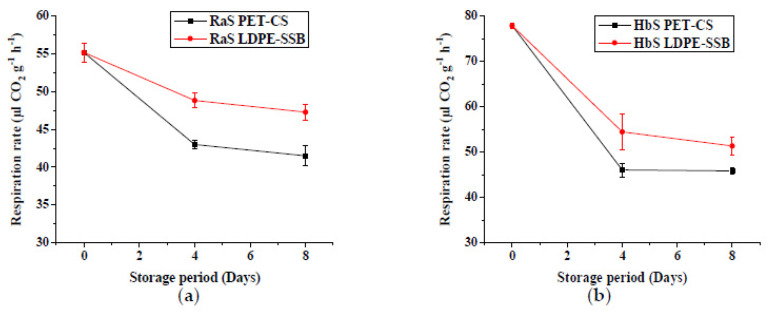
Effect of packaging on the respiration rate (µL CO_2_ g^−1^ h^−1^) in (**a**) radish microgreens (RaS) and (**b**) roselle microgreens (HbS) during storage at 5 °C. PET–CS: PET clamshell containers; LDPE–SSB: LDPE self-seal bags; data are means ± SD (*n* = 3).

The effect of packaging on the RR is represented in Figure 3. Respiration rates were significantly (*p* ≤ 0.05) affected by the type of packaging during storage, and PET–CS stored samples, maintaining lower RR throughout storage. A comparatively lower initial RR was obtained in RaS microgreens (55.2 µL CO_2_ g^−1^ h^−1^) than HbS microgreens (77.9 µL CO_2_ g^−1^ h^−1^). In both microgreens, a significant decrease (*p* ≤ 0.05) was noted in RR from days 0 to 4 of storage, and it remained relatively unchanged from the 4th day till the end of storage. Significantly lower RR was recorded in PET–CS (RaS: 41.5 µL CO_2_ g^−1^ h^−1^; HbS: 45.8 µL CO_2_ g^−1^ h^−1^) compared to LDPE–SSB (RaS: 47.3 µL CO_2_ g^−1^ h^−1^; HbS: 51.3 µL CO_2_ g^−1^ h^−1^) at the end of storage.

#### 3.1.3. Electrolyte Leakage (EL)

**Figure 4:**
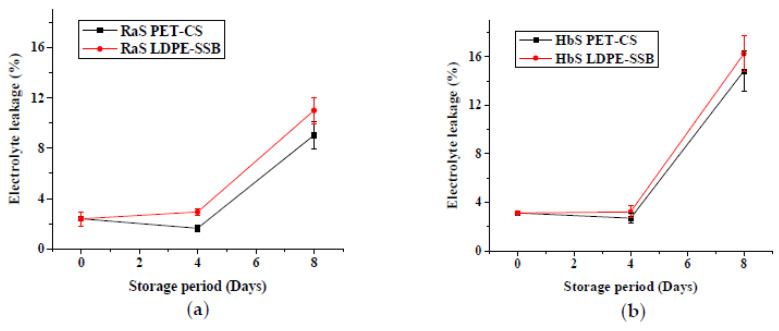
Effect of packaging on the electrolyte leakage (%) in (**a**) radish microgreens (RaS) and (**b**) roselle microgreens (HbS) during storage at 5 °C. PET–CS: PET clamshell containers; LDPE–SSB: LDPE self-seal bags; data are means ± SD (*n* = 3).

The initial values of EL of RaS and HbS microgreens were 2.4% and 3.1%, respectively (Figure 4). The EL remained relatively constant for up to 4 days. The values subsequently increased to 9% and 11% in RaS microgreens, and 14.8% and 16.3% in HbS microgreens, stored in PET–CS and LDPE–SSB packaging, respectively, on the 8th day of storage. PET–CS stored samples had lower EL compared to LDPE–SSB stored samples. However, the difference between the two packaging was significant (*p* ≤ 0.05) only in RaS microgreens at the end of storage.

#### 3.1.4. Instrumental Colour

**Figure 5:**
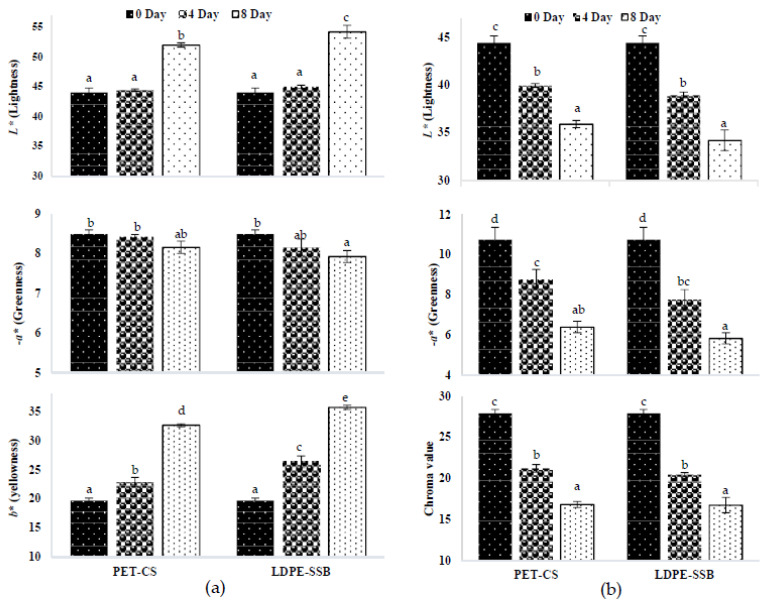
Effect of packaging on the colour coordinates, L* (lightness), −a* (greenness) and b* (yellowness) of (**a**) radish microgreens (RaS) and (**b**) roselle (HbS) microgreens during storage at 5 °C. PET–CS: PET clamshell containers; LDPE–SSB: LDPE self-seal bags; Different alphabets within the graph indicate a significant difference between packaging across storage at *p* ≤ 0.05; data are means ± SD (*n* = 3).

Instrumental colour was recorded to quantify the colour change in microgreens. Yellowing was the major discolouration seen in radish microgreens. Figure 5a represents the lightness (L*), degree of greenness (−a*), and yellowness (b*) of RaS microgreens during storage. At harvest, RaS microgreens had an average L* of 44.1, −a* of 8.5 and b* of 19.7. A significant increase in the L* coordinate was observed during storage with PET–CS having significantly lower L* value on the 8th day. No significant change was observed in −a* coordinate in PET–CS across storage, while on the 8th day, significantly lower −a* value was recorded in LDPE–SSB. Yellowing was observed in both types of packaging. At the end of the storage period, PET–CS RaS recorded significantly lower (32.6) b* values compared to LDPE–SSB samples (35.7). In HbS microgreens (Figure 5b), there was a significant reduction (*p* ≤ 0.05) in the L* value from 44.4 on day 0 to 35.9 in PET–CS, and 34.2 in LDPE–SSB on the 8th day. The −a* coordinate had a reduction from 10.8 on day 0 to 6.4 in PET–CS and 5.8 in LDPE–SSB after the 8th day of storage. Chroma value was calculated in HbS microgreens to trace the discolouration, as instead of yellowing, browning was observed. The chroma value decreased from 27.9 to 16.8 in both packaging across the storage. No significant difference was noted between the two packaging on the degree of discoloration of HbS microgreens.

#### 3.1.5. Microbial Quality

**Figure 6:**
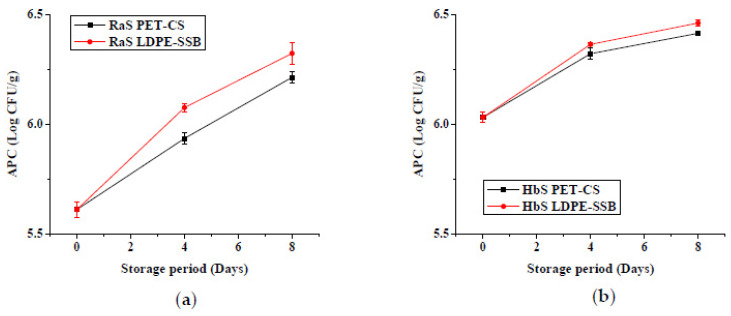
Effect of packaging on the aerobic plate count (APC; log CFU/g) in (**a**) radish microgreens (RaS) and (**b**) roselle microgreens (HbS) during storage at 5 °C. PET–CS: PET clamshell containers; LDPE–SSB: LDPE self-seal bags; data are means ± SD (*n* = 3).

**Figure 7:**
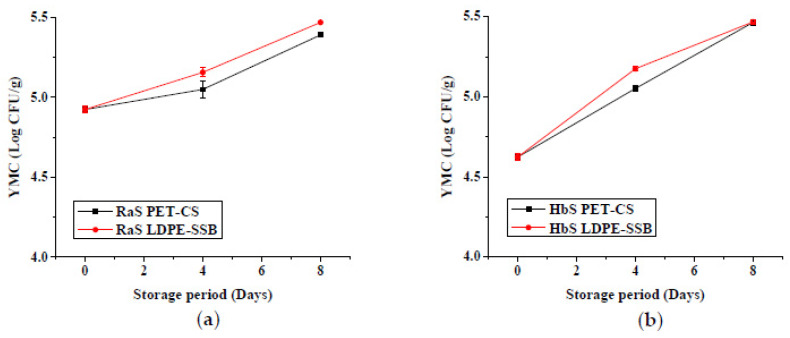
Effect of packaging on the yeast and mold count (YMC; log CFU/g) in (**a**) radish microgreens (RaS) and (**b**) roselle microgreens (HbS) during storage at 5 °C. PET–CS: PET clamshell containers; LDPE–SSB: LDPE self-seal bags; data are means ± SD (*n* = 3).

Changes in the aerobic plate count (APC) and yeast and mold count (YMC) in the two microgreens (Ras and HbS) during storage are given in Figure 6 and Figure 7, respectively. RaS microgreens had an initial APC and YMC of 5.6 and 4.9 log CFU/g, respectively. In the case of the HbS sample, an initial APC of 6 log CFU/g and YMC of 4.6 log CFU/g were recorded. The APC increased by 0.7 and 0.43 log in LDPE–SSB, while PET–CS sample showed a lower increase of 0.6 and 0.38 log at the end of storage in RaS and HbS microgreens, respectively. A lower increase in YMC was observed in PET–CS RaS (0.47 log) and HbS (0.67 Log), compared to LDPE–SSB RaS (0.55 log) and HbS (0.85 log) microgreens.

#### 3.1.6. Ascorbic Acid

The effect of packaging on the total (TAA), free (FAA), and dehydro-ascorbic acid (DHA) content along with the DHA/FAA ratio is depicted in Table 2. At harvest, HbS microgreens had higher TAA and FAA, and comparatively lower DHA/FAA ratio compared to RaS microgreens. As the storage period increased, there was a substantial reduction in FAA and TAA content in all the samples, with a concomitant increase in DHA. At the end of the storage period, no significant differences in AA levels were found with respect to the effect of packaging in RaS microgreens. However, roselle microgreens stored in the PET–CS package had significantly (*p* ≤ 0.05) lower DHA and DHA/FAA ratio compared to LDPE–SSB.

#### 3.1.7. Overall Acceptability and Marketability

The effect of packaging on the overall acceptability (OA) and marketability scores (MS) of RaS and HbS microgreens across storage is shown in Table 3. The detailed computation leading to the marketability scores is given in Table S1. At harvest, the OA for RaS and HbS microgreens were 8.3 and 8.4, respectively, and both had the highest MS of 5. A gradual reduction was observed in the OA during storage in both samples but remained within acceptable levels (>6.5). Overall, microgreens packed in PET–CS showed slightly higher consumer acceptability compared to LDPE-packaged ones in both the microgreens. However, this difference was not significant. On the 8th day, the highest OA was recorded in RaS PET–CS. HbS samples stored in PET–CS also had greater OA than LDPE–SSB stored samples. Marketability was not affected greatly by the type of packaging. All samples, except HbS LDPE–SSB, had an MS of 3 on the 8th day. Digital photographs depicting the effect of packaging on RaS and HbS microgreens are presented in Figure S1.

### 3.2. Comparative Effect of Edible Coating Techniques on Postharvest Quality and Shelf Life of Radish (RaS) and Roselle (HbS) Microgreens

#### 3.2.1. Physiological Loss in Weight

**Figure 8:**
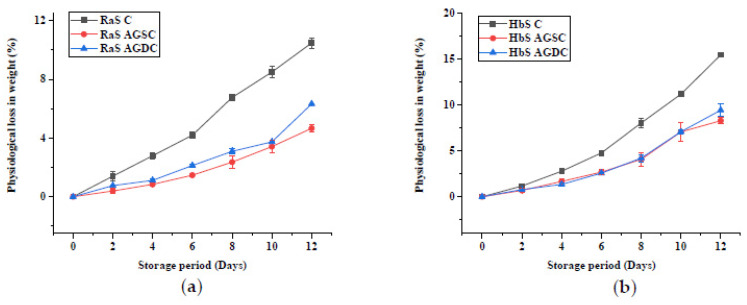
Effect of edible coating technique on the physiological loss in weight (%) in (**a**) radish microgreens (RaS) and (**b**) roselle microgreens (HbS) during storage at 5 °C; C—control, AGSC—Aloe gel spray-coated, AGDC—Aloe gel dip-coated; data are means ± SD (*n* = 3).

The effect of the edible coating technique on the PLW is shown in Figure 8. A significantly (*p* ≤ 0.05) lower PLW was noted in all the coated samples in both RaS and HbS microgreens compared to the respective controls throughout storage. No significant difference was observed between spray- and dip-coated (AGDP) microgreens, except on the 12th day. The values in coated samples were lower than uncoated samples with RaS C microgreens recording a PLW of 10.5%, while HbS C had a relatively higher PLW of 15.5%. Least PLW of 4.7% and 8.3% were obtained in spray-coated Aloe gel (AGSC) RaS and HbS microgreens, respectively. These values were lower than that of the PLW recorded in AGDP RaS (6.3%) and HbS (9.4%) microgreens at the end of the storage period.

#### 3.2.2. Respiration Rate

**Figure 9:**
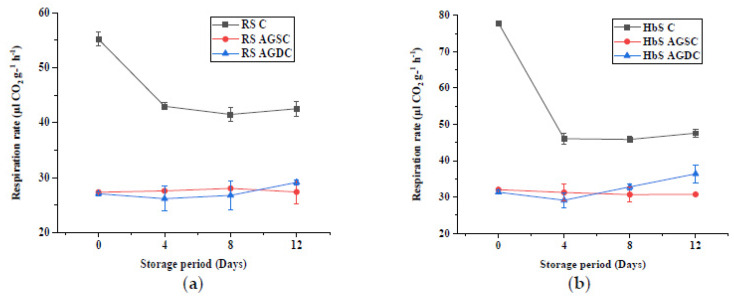
Effect of edible coating technique on the respiration rate (µL CO_2_ g^−1^ h^−1^) in (**a**) radish microgreens (RaS) and (**b**) roselle microgreens (HbS) during storage at 5 °C. C—control uncoated, AGSC—Aloe gel spray-coated, AGDC—Aloe gel dip-coated; data are means ± SD (*n* = 3).

The effect of the edible coating technique on the RR of microgreens is represented in Figure 9. In both RaS and HbS microgreens, significantly lower initial RR was recorded in AGSC (RaS: 27.4 µL CO_2_ g^−1^ h^−1^; HbS: 32 µL CO_2_ g^−1^ h^−1^) and AGDC (RaS: 27.1 µL CO_2_ g^−1^ h^−1^; HbS: 31.3 µL CO_2_ g^−1^ h^−1^) microgreens compared to C (RaS: 55.2 µL CO_2_ g^−1^ h^−1^; HbS: 77.9 µL CO_2_ g^−1^ h^−1^). The RR of C samples showed a pronounced decrease during storage up to the 4th day and remained relatively constant till the end of storage with values of 42.5 µL CO_2_ g^−1^ h^−1^ in RaS and 47.6 µL CO_2_ g^−1^ h^−1^ in HbS microgreens on the 12th day. No significant differences in RR were observed between the two edible coating techniques during the storage of RaS microgreens. RaS AGDC maintained slightly lower values compared to AGSC for up to 8 days. However, the initial RR of the coated RaS microgreens remained relatively unchanged up to the 12th day of storage in AGSC (27.4 µL CO_2_ g^−1^ h^−1^) and AGDC (29.2 µL CO_2_ g^−1^ h^−1^) samples. With respect to HbS microgreens, a relatively lower initial RR was obtained in the AGDC sample compared to the AGSC sample on days 0 and 4 of storage; however, this difference was not significant. A gradual increase was noted from the 4th day in the AGDC sample, ending with a significantly higher (*p* ≤ 0.05) RR of 36.4 µL CO_2_ g^−1^ h^−1^compared to AGSC (30.7 µL CO_2_ g^−1^ h^−1^) sample on the 12th day of storage. These values were significantly lower (*p* ≤ 0.05) than HbS C.

#### 3.2.3. Electrolyte Leakage

**Figure 10:**
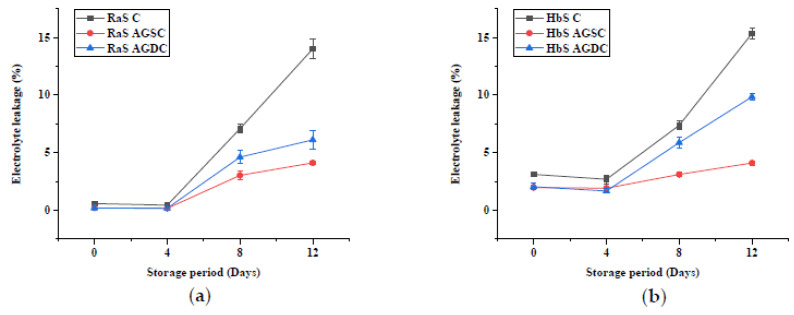
Effect of the edible coating technique on the electrolyte leakage (%) in (**a**) radish microgreens (RaS) and (**b**) roselle microgreens (HbS) during storage at 5 °C; C—control uncoated, AGSC—Aloe gel spray-coated, AGDC—Aloe gel dip-coated; data are means ± SD (*n* = 3).

Figure 10 shows the effect of *Aloe vera* gel spray and dip coating on the electrolyte leakage (EL) of RaS and HbS microgreens. Negligible EL was noted in the case of RaS microgreens from 0 to 4 days of storage. The presence of edible coating reduced the initial EL (0.2% in both AGSC and AGDC RaS) compared to RaS C (0.6%). In HbS microgreens too, AGDC and AGSC samples recorded lower EL of 2% and 1.98%, respectively, compared to the HbS C sample (3.1%). All samples had an initial reduction in the EL, followed by different degrees of increase after the 4th day. On the 12th day of storage, the least increase in EL was observed in RaS and HbS AGSC microgreens (4.1% both) compared to the respective controls (RaS C—14% and HbS C—15.3%) and the AGDC samples. HbS AGDC microgreens had a sharp increase after the 4th day, resulting in an EL of 9.9% at the end of storage. The less pronounced increase was noted in AGDC RaS microgreens with a final value of 6.1%. With respect to EL, the spray-coating technique showed better results compared to dip-coating in the studied microgreens.

#### 3.2.4. Instrumental Colour

**Figure 11:**
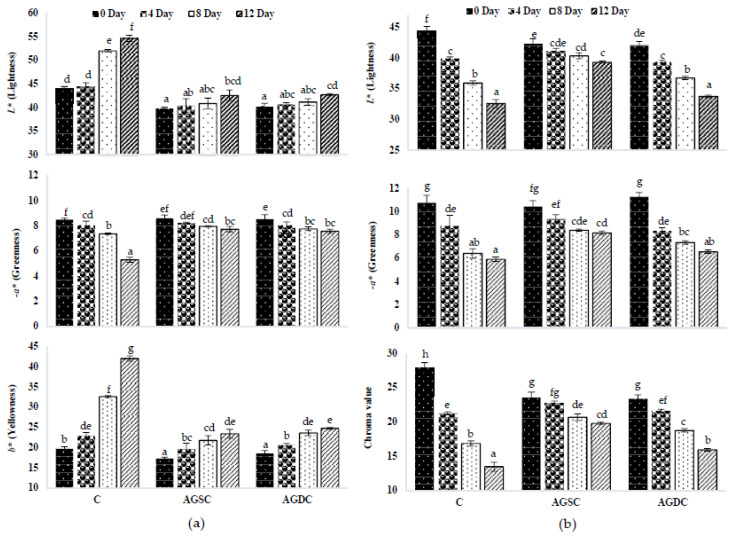
Effect of edible coating technique on the colour coordinates, L* (lightness) −a* (greenness) and b* (yellowness) of (**a**) radish microgreens (RaS) and (**b**) roselle microgreens (HbS) during storage at 5 °C; C—control uncoated, AGSC—Aloe gel spray-coated, AGDC—Aloe gel dip-coated; Different alphabets within the graph indicate a significant difference between packaging across storage at *p* ≤ 0.05; data are means ± SD (*n* = 3).

The effect of the edible coating technique on the instrumental colour of RaS and HbS microgreens during storage is represented in Figure 11a,b, respectively. In RaS microgreens, L* coordinate in C had a pronounced increase during storage (44.4 to 54.7). However, a very slight increase occurred in the L* coordinate of the Aloe gel-coated sample (AGSC: 39.8 to 42.6 and AGDC: 40.2 to 42.8) during the storage period. Aloe gel-coated RaS microgreens had minimal change in the −a* coordinate during storage (AGSC: 8.6 to 7.7; AGDC: 8.5 to 7.6), while the control sample had a significant reduction (*p* ≤ 0.05) from 8.5 to 5.3 at the end of the 12th day. With respect to the b* coordinate, the highest increase was noted in the C sample (19.7 to 42.1). A significantly lower degree of increase was noted in AGSC (17.3 to 23.4) and AGDC (18.5 to 24.7) samples from 0 to 12th day in terms of b*. No significant difference was noted between the coating techniques on the colour coordinates with respect to RaS microgreens. In the case of HbS microgreens, a significant reduction was observed in L* coordinate in C sample (44.4 to 32.6) at the end of storage. AGSC maintained significantly higher (*p* ≤ 0.05) L* value (39.3) than C and AGDC (33.8). AGSC maintained the highest −a* value of 8.2 at the end of the storage, with significantly lower (*p ≤* 0.05) values in AGDC (6.5) and C (5.9). The chroma values followed the same trend, with AGSC (23.6 to 19.8) having a significantly lower reduction (*p ≤* 0.05) compared to AGDC (23.4 to 15.9) and C (27.9 to 13.5) on the 12th day of storage.

#### 3.2.5. Microbial Quality

**Figure 12:**
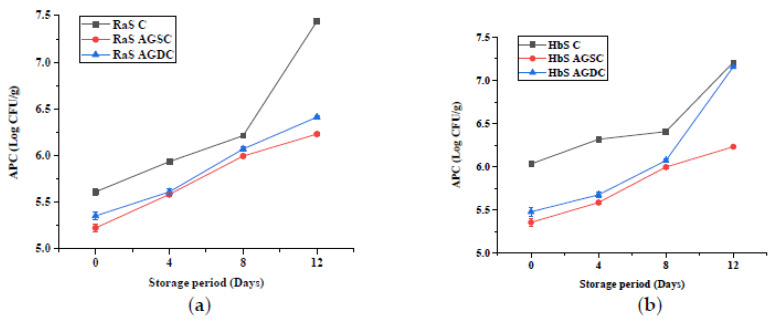
Effect of edible coating technique on the aerobic plate count (APC; log CFU/g) in (**a**) radish microgreens and (**b**) roselle microgreens during storage at 5 °C; C—control uncoated, AGSC—Aloe gel spray-coated, AGDC—Aloe gel dip-coated; data are means ± SD (*n* = 3).

**Figure 13:**
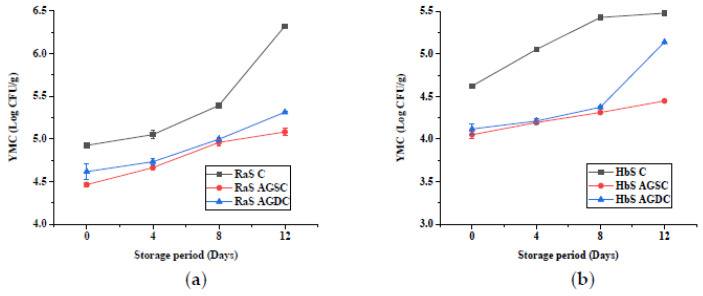
Effect of edible coating technique on yeast and mold count (YMC; log CFU/g) in (**a**) radish microgreens (RaS) and (**b**) roselle microgreens (HbS) during storage at 5 °C; C—control uncoated, AGSC—Aloe gel spray-coated, AGDC—Aloe gel dip-coated; data are means ± SD (*n* = 3).

The effect of the edible coating technique on the microbial quality (APC and YMC) of RaS and HbS microgreens is represented in Figure 12 and Figure 13, respectively. Significantly lower (*p ≤* 0.05) initial APC was recorded in both Aloe gel-coated RaS (AGSC: 5.2 log CFU/g; AGDC: 5.5 log CFU/g) and HbS (AGSC: 5.4 log CFU/g; AGDC: 5.5 log CFU/g) microgreens compared to control (RaS C: 5.6 log CFU/g; HbS C: 6.0 log CFU/g). In both microgreens, AGSC samples maintained significantly lower (*p ≤* 0.05) APC than control throughout the storage period. In RaS microgreens, significantly lower APC was observed in AGSC compared to AGDC treatments on 0 and 12th day of storage. In the case of HbS, AGSC samples maintained significantly lower (*p ≤* 0.05) APC than AGDC throughout storage. The YMC followed a similar trend to the APC. At the end of storage, the RaS C sample had a pronounced increase (*p ≤* 0.05) in the YMC of 1.4 log. In both microgreens, AGSC maintained relatively lower YMC than AGDC throughout storage, but this change was not significant in RaS. In HbS microgreens, significantly lower (*p ≤* 0.05) YMC was recorded in AGSC on the 12th day of storage compared to AGDC, with values of 4.5 and 5.1 log CFU/g, respectively. Both the treatments, however, recorded a larger and significantly lower YMC than the control uncoated samples.

#### 3.2.6. Ascorbic Acid

Table 4 shows the effect of the edible coating technique on the TAA, FAA, and DHA contents along with the DHA/FAA ratio across storage. The results show significantly higher (*p ≤* 0.05) initial contents of TAA and FAA in AGSC and AGDC samples than the C in both RaS and HbS microgreens. A gradual reduction in the TAA and FAA contents occurred in all samples, accompanied by an increase in the DHA/FAA ratio. Among the edible coating techniques, the highest content of TAA was recorded in AGSC samples of both microgreens throughout storage. However, the differences between AGSC and AGDC were not significant. Interestingly, the DHA/FAA ratio showed a significant difference (*p ≤* 0.05) between RaS AGSC and RaS AGDC on the 12th day of storage. In both microgreens, AGSC and AGDC DHA/FAA ratios were significantly lower (*p ≤* 0.05) than the controls. Maximum TAA loss was noted in HbS C microgreens (63%), followed by RaS C microgreens (42%) at the end of 12-day storage.

#### 3.2.7. Overall Acceptability and Marketability

The effect of the edible coating technique on the OA and MS of RaS and HbS microgreens during storage is given in Table 5. The detailed computation leading to the marketability scores is given in Table S2. Comparatively higher initial OA was obtained in Aloe gel-coated (AGSC and AGDC) samples compared to control in both microgreens. With increasing storage period, a reduction in the OA occurred in all samples, with C samples rapidly losing their marketability (score of 3) on the 8th day of storage. RaS and HbS AGSC microgreens maintained the highest OA till the end of storage. In RaS microgreens, AGDC maintained equally good marketability, with a slightly lower OA score. However, in the case of HbS microgreens, the AGDC sample lost its marketability by the 12th day of storage. Digital photographs depicting the effect of edible coating on RaS and HbS microgreens are presented in Figure S2.

#### 3.2.8. Scanning Electron Microscope (SEM) Image Analysis

**Figure 14:**
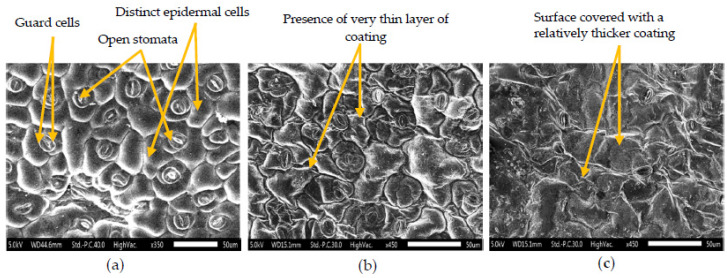
SEM images of roselle microgreens (**a**) uncoated, (**b**) Aloe gel spray-coated, and (**c**) Aloe gel dip-coated.

The SEM images showing the surface morphology of uncoated control and Aloe gel-coated HbS microgreens with spray and dip techniques are presented in Figure 14. Stomatal apertures, guard cells and epidermal cells were distinctly visible on the surface of uncoated control. The SEM image of AGSC revealed the presence of a well-distributed thin coating forming a semi-permeable barrier showing indistinct stomata and epidermal cells. In the case of AGDC, the surface was covered with a relatively thicker and non-uniform coating showing very few stomatal apertures and no distinct epidermal cells visible.

## 4. Discussion

Physiological loss in weight is a natural process of catabolism brought about by enzymes, and accelerated by mechanical injuries like cutting and slicing, in fresh horticultural produce [33]. The decrease in weight may be attributed to respiration and other senescence-related metabolic processes during storage [34]. In a study carried out in macro-perforated BOPP packaging, a weight loss of 60% was reported in traditional leafy vegetables stored for 4 days at 10 °C [35]. The much lower weight loss in the present study may be attributed to the lower storage temperature (5 °C). The better performance of PET-CS could be attributed to the lower water vapour permeability of the packaging material compared to LDPE [36]. Macro-perforated packaging, commonly preferred in commercial set-ups, is beneficial in reducing off-flavour of fresh produce [8], as it prevents anaerobic atmosphere, even under temperature abuse situations [37]. However, it leads to higher fresh weight losses and requires other strategies to minimize the PLW. Considerable reduction in the PLW was obtained with Aloe gel-based edible coating in the present study. The spray coating technique was found to be as good or even better than the dip-coating in minimising losses in fresh weight. This could possibly be due to uniformity of the coating and immediate packaging of the microgreens after harvest in the case of AGSC compared to the time delay in allowing the coating to dry in the case of AGDC. Significant reduction in weight loss was also reported in Aloe gel-treated fresh-cut lotus root slices with weight losses of ~6% in 25% AG and ~ 4% in 50% AG compared to ~10% in control on the 8th day of storage at 5 °C [16].

Based on the classification of vegetables given by Kader and Salveit [38], according to their relative respiration rates and degree of perishability, the studied microgreens can be categorized as fresh produce with very high respiration rates (>30 µL CO_2_ g^−1^ h^−1^) when stored at 5 °C. Such high respiration rates limit their shelf life [3], and their storage requires packaging with good O_2_ permeability to prevent anaerobic conditions and off-odour development [39]. Thus, perforated packaging material, generally used for salad crops, is expected to enhance the post-harvest performance of microgreens [2]. The initial respiration rates of the studied microgreens were found to be comparable to that of young leaves of parsley (~ 50 µL CO_2_ g^−1^ h^−1^) [40] and asparagus (96 µL CO_2_ g^−1^ h^−1^) [38] when stored at 5 °C. Very high respiration rates (99–111 µL CO_2_ g^−1^ h^−1^) were also reported in arugula, radish and red cabbage microgreens when stored at 4 °C [5]. The decrease in the respiration rate during storage may be attributed to the depletion of carbohydrate reserves, which function as substrates for the respiration process [41]. The lower RR obtained PET-CS could be due to the lower O_2_ permeability compared to LDPE-SSB packaging [42]. In addition, the RR was also species-specific as they undergo senescence due to differences in the surface area to volume ratio and their surface characteristics (e.g., cuticle thickness, stomata, lenticels) [38]. Interestingly, the treatment of microgreens with *Aloe vera* gel coating considerably reduced the RR throughout storage in both samples with AGDC having a slightly lower respiration rate than AGSC initially. This was evidenced by the SEM images, which showed partial covering of the stomatal apertures in the case of AGSC, while AGDC images showed the presence of unclear stomata only in certain regions of the surface, indicating a thicker coating. However, as storage progressed, AGDC samples showed a comparatively higher RR than AGSC at the end of storage. This could be due to relatively thicker coating in AGDC samples leading to excess inhibition of O_2_ and more production of CO_2_ as a result of anaerobic respiration [42]. Similar results were obtained by Nasrin et al., who observed a significant reduction in the respiration rate of Aloe gel-coated strawberries compared to uncoated ones [43].

Electrolyte leakage is a common index of senescence that reflects the deterioration of membranes caused by physiological stress or mechanical injury [44]. Electrolyte leakage has been linked to the postharvest quality and shelf life of microgreens [3,4,31]. Comparable EL was reported in previous studies in broccoli microgreens [27,28]. Our observation with respect to the effect of packaging on EL is in line with the results of Xiao et al. [31], who also found no significant effect of packaging on the EL of radish microgreens. However, the comparatively lower EL obtained in PET–CS could be due to the nature of packaging. PET–CS being a thicker and sturdier packaging than LDPE–SSB could have protected against mechanical injuries during handling, leading to lesser injured plant tissues, hence lower EL. The lower EL in Aloe gel treated microgreens (AGSC and AGDC) compared to the control could be due to improved cell integrity, as shown in an earlier study on Aloe gel-coated bell pepper [45]. A significant reduction in the EL was also observed in Aloe-treated lotus root slices compared to uncoated ones [16]. In another study, chitosan treated fruit had a more structured cell arrangement than the uncoated cell, which was characterized by cellular plasmolysis, loss of turgor pressure as senescence progresses [46]. Between the edible coating treatments, AGSC of both samples had lower EL than AGDC. In an earlier study, postharvest dip treatment, in general, was found to have accelerated tissue senescence and quality deterioration due to mechanical damage, which can incur during spinning and drying after the dip [28].

The colour of microgreens is an important factor affecting their visual appearance. While RaS microgreens had an increase in lightness, corresponding to the increase in yellowing, HbS samples had a decrease in lightness with storage, which could be due to browning of the leaves. Both microgreens had a reduction in greenness during storage when stored in PET–CS and LDPE–SSB. The type of packaging did not have any significant effect on the colour coordinates of the microgreens. However, during senescence, the pattern of change in colour can differ among different vegetables [47]. RaS microgreens exhibited yellowing of leaves during storage, as indicated by an increase in the b* coordinate. A similar trend was observed by Supapvanich et al. [48] in sweet leaf bush during 8 days of storage. This loss of greenness could be attributed to the breakdown of chlorophyll molecules by the chlorophyll-degrading enzymes such as chlorophyll oxidase, chlorophyll peroxidase, and chlorophyllase, revealing the pre-existing yellow carotenoid pigments [49]. HbS microgreens, on the other hand, showed evidence of browning during the storage period, corresponding with the loss of overall chromaticity of HbS microgreens. Earlier studies have reported changes in colour coordinates correlating with the incidence of browning [50,51]. Browning is generally considered to be caused by a range of endogenous phenolic compounds containing an *o*-dihydroxy group that gets oxidised to the corresponding *o*-quinone by oxidising enzymes in the presence of oxygen, leading to the formation of brown pigments (melanin) [52]. Roselle microgreens indeed had the highest content of total phenolics among ten microgreens analysed [53]. However, the application of edible coating (AGSC and AGDC) helped to reduce discolouration and retained the greenness of microgreens. The gas-barrier function of edible coating could retard loss of colour components and enzymatic oxidation, protecting fresh produce from discoloration and texture softening during storage [35]. In an earlier study on minimally processed vegetables, the application of chitosan coating exhibited higher chroma values throughout storage compared to uncoated samples [22]. In the case of HbS microgreens, the overall chromaticity was better maintained in AGSC samples, as evidenced by a lower incidence of browning compared to HbS AGDC samples, observed during the latter part of storage. A similar reduction in the degree of browning was also observed in fresh-cut lotus root slices coated with 50% Aloe vera gel [16]. This could be attributed to the higher EL recorded in the AGDC HbS samples. EL is an indicator of senescence due to physical damage/wounding. This is expected to have occurred during the dip-coating process, leading to an increased rate of biochemical reactions responsible for changes in colour (browning) [54]. It is also important to note that HbS microgreens have softer and thinner cotyledonary leaves compared to RaS microgreens, making it comparatively more susceptible to mechanical injuries than the latter.

Microgreens are generally characterized by high moisture and nutrient contents, which create an environment conducive for the growth of microorganisms. The initial APC and YMC were comparable to those obtained in buckwheat microgreens [4], broccoli and chicory microgreens [55]. Between the two packaging, the significantly lower microbial count in PET–CS could be attributed to the sturdiness of the packaging, which could have reduced chances of mechanical injuries, hence limiting the proliferation of the microorganisms. This packaging also helped better retention of fresh weight. Weight loss is characterized by an increase in intercellular spaces within the plant tissue, which can facilitate the entry of microorganisms into cells [46]. In the phase II study, the increase in microbial load in the control uncoated samples during storage corresponded with an increase in weight loss and electrolyte leakage of the microgreens. Leakage of juices and sugars from damaged plant tissues has been reported to favour microbial growth [56]. *Aloe vera* gel coating significantly reduced the APC and YMC in both microgreens during storage. *Aloe vera* gel is known to contain several compounds such as saponins, tannins, flavonoids, and terpenoids, which are responsible for its antimicrobial activity [57]. The results obtained were comparable with the effects of *Aloe vera* gel coating in sweet cherry [58] and apple slices [59], which showed a reduction in the mesophilic aerobic bacteria and yeast and mold counts during storage. With respect to the edible coating techniques, the higher microbial load recorded in AGDC compared to AGSC samples at the end of storage could be attributed to the higher EL, RR and/or contamination during the postharvest dip.

Ascorbic acid is the vitamin that is most sensitive to destruction during the storage of fresh commodities and hence can be used as a chemical indicator of shelf life quality [60]. Similar contents of total, free and dehydro-ascorbic acid were reported in microgreens in an earlier study [61]. In the current study, the macro-perforated packaging increased the availability of oxygen, which could have favoured ascorbate oxidase, the enzyme responsible for the oxidation of FAA to DHA [62]. Loss of fresh weight has been associated with rapid ascorbic acid degradation as well [63]. However, no significant difference was obtained between the two forms of packaging, which could be due to the macro-perforated nature of the packaging. The reduction of TAA and FAA and the accumulation of DHA during storage were also observed in fresh-cut spinach [64] and minimally processed lettuce [65]. A significantly higher ascorbic acid content was obtained in Aloe gel-coated samples compared to uncoated control. This could be attributed to the presence of ascorbic acid in the inner gel [66]. This could be a promising way to enhance the ascorbic acid content of the microgreens using plant-based bio-treatments like *Aloe vera* gel. Similar observations were reported earlier in litchi fruit [21], raspberry fruits [67] and tomato [68] coated with *Aloe vera* gel. At the end of storage, AG treatments exhibited lower reductions in ascorbic acid in RaS microgreens (AGSC: 34.5%; AGDC: 34.4%) and HbS microgreens (AGSC: 45.6%; AGDC: 50.8%) compared to respective controls (41.6% and 62.5%). The oxidative loss of ascorbic acid could have been reduced by the presence of the protective coating [69]. Between the two edible coating techniques, a significantly lower DHA/FAA ratio was observed in AGSC compared to AGDC at the end of storage. This could be attributed to the higher levels of oxidative stress as indicated by higher RR, and injury to the membranes during the dip-coating treatment indicated by higher EL, leading to greater activity of ascorbic acid degrading enzymes [70] in AGDC samples.

Overall acceptability of the produce directly influences their marketability. The relatively higher OA in PET–CS could be attributed to the better appearance and texture of the microgreens packaged in PET–CS compared to that in LDPE. This could be due to the sturdy nature of the PET–CS package, which conferred better protection to the microgreens during storage. Also, clamshell containers are known to provide efficiency in terms of shipping and merchandising [71]. On the other hand, LDPE–SSB stored samples could be more prone to mechanical injuries during handling due to the flexible nature of the packaging. The loss of OA during storage is related to aging processes and senescence [60], as observed by an increase in PLW, RR, and EL, leading to a reduction in marketability. HbS LDPE–SSB samples lost their marketability on the 6th day of storage, while PET–CS stored was marketable up to the 8th day of storage. The comparatively sturdier nature of RaS microgreens enabled it to better withstand the effect of handling and thus showed a marketability of 8 days in both packaging. Application of *Aloe vera* gel coating resulted in better retention of freshness and added a glossy sheen on the surface of microgreens resulting in better overall acceptability compared to the uncoated samples. It also reduced the pungent taste of RaS microgreens. HbS AGDC lost its shelf marketability by the 12th day of storage due to browning of leaves and lower consumer acceptability, while HbS AGSC was marketable beyond 12 days. In the case of RaS samples, though relatively higher OA was recorded in AGSC than AGDC, both treatments were marketable beyond 12 days. It could be hypothesised that the two techniques are equally effective in retaining the freshness of the microgreens, as indicted by a much lower degree of witling. In samples prone to browning such as HbS, a thinner coating delivered by the spray technique could be more advantageous.

## 5. Conclusions

In conclusion, macro-perforated PET-CS was found to be a comparatively better packaging than LDPE–SSB for postharvest quality maintenance during the storage of RaS and HbS microgreens. Though PET–CS would be commercially preferred as a rigid packaging during long-distance transportation, LDPE–SSB could also be used as an economical alternative in short distance markets and for sturdier microgreens. *Aloe vera* gel edible coating acted as a primary packaging and helped to overcome the drawbacks of macro-perforated packaging and significantly enhanced the postharvest quality and shelf life of the studied microgreens. Aloe gel coated samples also had ~40% to 70% higher total ascorbic acid content and maintained 2- to 3-fold lower DHA/FAA ratios compared to uncoated ones. Aloe gel coating is an eco-friendly and sustainable alternative to chemical pre-treatments for shelf-life quality enhancement in the studied microgreens. The spray-coating technique performed similar to or better than the dip-coating technique. It was found to be a superior option as it helped to circumvent the drawbacks of the dip-coating technique, such as dilution of coating solution and risk of contamination. The AGSC technique also offers the advantages of uniform coating, lesser handling of microgreens and lower coating solution requirement, leading to reduced cost (approx. 10-fold less). In addition, it is also amenable to large scale setups, making it a promising preharvest treatment for enhancing the postharvest quality and shelf life of radish and roselle microgreens. The AGSC technique, along with PET–CS, has potential for applications in other microgreens and fresh-cut produce. Future work will consider the use of biodegradable packaging along with edible coating as a total sustainable packaging approach for premium produce like microgreens. In addition, edible coating formulated for nutrient enrichment of high-value microgreens is also under evaluation.

## Figures and Tables

**Table 1:** Percentage loss of saleability and marketability score for shelf life quality assessment.

Loss of Saleability (%)	Marketability Score
0 < 10	5
10 < 19	4
20 < 29	3
30 < 39	2
>40	1

**Table 2:** Effect of packaging on the free (FAA), dehydro (DHA) and total ascorbic acid (TAA) contents of radish and roselle microgreens across storage.

Packaging	Storage Period (Days)	Ascorbic Acid (mg/100 g)			
		FAA	DHA	TAA	DHA/FAA Ratio
		*Radish Microgreens*			
PET–CS	0	67.83 ± 0.69 ^d^	6.15 ± 0.79 ^a^	73.98 ± 3.82 ^d^	0.091 ^a^
	4	55.08 ± 1.03 ^c^	10.85 ± 1.19 ^b^	65.93 ± 2.54 ^c^	0.197 ^b^
	8	38.29 ± 0.68 ^a^	16.30 ± 0.78 ^cd^	54.59 ± 2.46 ^a^	0.426 ^d^
LDPE–SSB	0	67.83 ± 0.69 ^d^	6.15 ± 0.79 ^a^	73.98 ± 3.82 ^d^	0.091 ^a^
	4	49.96 ± 2.26 ^b^	14.08 ± 2.61 ^c^	64.04 ± 2.51 ^bc^	0.284 ^c^
	8	38.40 ± 0.76 ^a^	18.98 ± 0.88 ^d^	57.38 ± 1.92 ^ab^	0.495 ^d^
		*Roselle Microgreens*			
PET–CS	0	98.71 ± 0.44 ^d^	7.92 ± 0.51 ^a^	106.62 ± 1.77 ^d^	0.080 ^a^
	4	52.58 ± 1.52 ^c^	15.14 ± 1.75 ^b^	70.01 ± 3.02 ^c^	0.289 ^b^
	8	28.66 ± 0.84 ^b^	20.58 ± 2.04 ^c^	49.24 ± 2.21 ^a^	0.718 ^c^
LDPE–SSB	0	98.71 ± 0.44 ^d^	7.92 ± 0.51 ^a^	106.62 ± 1.77 ^d^	0.080 ^a^
	4	48.92 ± 3.18 ^c^	18.80 ± 3.67 ^bc^	62.00 ± 0.99 ^b^	0.390 ^b^
	8	24.22 ± 1.78 ^a^	25.51 ± 1.91 ^d^	49.73 ± 2.00 ^a^	1.061 ^d^

PET–CS: PET clamshell containers; LDPE–SSB: LDPE self-seal bags. Different alphabets within the same column for each microgreen indicate significant difference at *p* ≤ 0.05 according to the Tukey HSD test; data are means ± SD (*n* = 4).

**Table 3:** Effect of packaging on the overall acceptability and marketability score of radish (RaS) and roselle (HbS) microgreens during storage at 5 °C.

Packaging	Storage Period (Days)	Radish Microgreens		Roselle Microgreens	
		OA	MS	OA	MS
PET–CS	0	8.3 ± 0.5 ^c^	5	8.4 ± 0.5 ^c^	5
	4	7.8 ± 0.4 ^b^	4	7.6 ± 0.6 ^b^	4
	8	7.0 ± 0.4 ^a^	3	6.6 ± 0.7 ^a^	3
LDPE–SSB	0	8.3 ± 0.5 ^c^	5	8.4 ± 0.5 ^c^	5
	4	7.5 ± 0.5 ^b^	4	7.4 ± 0.5 ^b^	4
	8	6.7 ± 0.7 ^a^	3	6.2 ± 0.7 ^a^	2

PET–CS: PET clamshell containers; LDPE–SSB: LDPE self-seal bags; OA: overall acceptability; MS: marketability score. Different alphabets within the same column indicate significant difference at *p* ≤ 0.05 according to the Tukey HSD test; data are means ± SD (*n* = 2).

**Table 4:** Effect of edible coating technique on the free (FAA), dehydro (DHA) and total ascorbic acid (TAA) contents of radish and roselle microgreens across storage.

Treatments	Storage Period (Days)	Ascorbic Acid (mg/100 g)			
		FAA	DHA	TAA	DHA/FAA Ratio
*Radish Microgreens*					
RaS C	0	78.56 ± 0.37 ^g^	6.63 ± 0.43 ^a^	85.19 ± 1.08 ^d^	0.084 ^ab^
	4	56.40 ± 1.13 ^d^	12.54 ± 1.30 ^bc^	68.94 ± 1.68 ^c^	0.223 ^abc^
	8	42.22 ± 1.34 ^b^	17.16 ± 1.54 ^de^	59.38 ± 2.92 ^b^	0.408 ^de^
	12	26.41 ± 2.41 ^a^	23.32 ± 2.78 ^f^	49.74 ± 1.97 ^a^	0.899 ^f^
RaS AGSC	0	99.88 ± 0.51 ^j^	8.16 ± 0.59 ^a^	108.04 ± 4.52 ^f^	0.082 ^a^
	4	83.45 ± 1.30 ^h^	9.68 ± 1.50 ^ab^	93.13 ± 3.55 ^e^	0.116 ^abc^
	8	70.97 ± 2.02 ^f^	12.79 ± 1.75 ^bc^	83.76 ± 2.91 ^d^	0.181 ^abc^
	12	56.25 ± 2.19 ^d^	14.55 ± 2.53 ^cd^	70.81 ± 3.36 ^c^	0.261 ^cd^
RaS AGDC	0	94.07 ± 0.73 ^i^	8.31 ± 0.84 ^a^	102.38 ± 1.47 ^f^	0.088 ^ab^
	4	72.90 ± 0.90 ^f^	12.38 ± 1.04 ^bc^	85.29 ± 3.24 ^d^	0.170 ^abc^
	8	63.49 ± 0.78 ^e^	15.34 ± 0.90 ^cd^	78.84 ± 1.88 ^d^	0.242 ^bc^
	12	47.07 ± 0.78 ^c^	20.12 ± 0.90 ^ef^	67.18 ± 0.98 ^c^	0.428 ^e^
*Roselle Microgreens*					
HbS C	0	107.67 ± 3.60 ^h^	7.40 ± 1.26 ^a^	115.07 ± 2.68 ^g^	0.069 ^a^
	4	50.21 ± 1.14 ^c^	15.00 ± 3.19 ^c^	65.19 ± 5.09 ^bc^	0.357 ^cd^
	8	31.97 ± 0.51 ^b^	16.29 ± 0.59 ^cd^	48.26 ±1.12 ^a^	0.510 ^d^
	12	23.45 ± 2.44 ^a^	19.66 ± 2.82 ^de^	43.11 ± 3.06 ^a^	0.858 ^e^
HbS AGSC	0	127.43 ± 0.84 ^j^	6.93 ± 0.97 ^a^	134.36 ± 6.81 ^h^	0.054 ^a^
	4	83.63 ± 0.57 ^g^	9.74 ± 0.57 ^ab^	93.37 ± 4.01 ^f^	0.116 ^b^
	8	67.41 ± 1.83 ^e^	14.69 ± 1.58 ^bcd^	82.10 ± 3.24 ^def^	0.219 ^abc^
	12	52.76 ± 2.02 ^c^	20.27 ± 1.75 ^de^	73.03 ± 7.49 ^bcd^	0.386 ^cd^
HbS AGDC	0	119.99 ± 1.76 ^i^	6.59 ± 1.53 ^a^	126.58 ± 2.67 ^h^	0.055 ^a^
	4	75.48 ± 1.40 ^f^	10.57 ± 1.22 ^abc^	86.05 ± 1.79 ^df^	0.140 ^ab^
	8	58.61 ± 1.19 ^d^	19.33 ± 1.03 ^de^	77.93 ± 1.81 ^cde^	0.330 ^bcd^
	12	49.19 ± 1.65 ^c^	23.84 ± 1.43 ^e^	62.30 ± 2.60 ^b^	0.486 ^d^

C—uncoated control; AGSC—Aloe gel spray-coated; AGDP—Aloe gel dip-coated; different alphabets within the same column indicate significant difference at *p* ≤ 0.05, according to Tukey HSD test; data are means ± SD (*n* = 4).

**Table 5:** Effect of edible coating techniques on the overall acceptability and marketability of radish and roselle microgreens during storage at 5 °C.

Edible Coating Technique	Storage Period (Days)	Radish Microgreens		Roselle Microgreens	
		OA	MS	OA	MS
C	0	8.3 ± 0.5 ^f^	5	8.4 ± 0.5 ^f^	5
	4	7.8 ± 0.4 ^de^	4	7.6 ± 0.6 ^cde^	4
	8	7.0 ± 0.4 ^b^	3	6.6 ± 0.7 ^b^	3
	12	6.4 ± 0.6 ^a^	2	5.8 ± 0.5 ^a^	1
AGSC	0	8.4 ± 0.5 ^f^	5	8.5 ± 0.5 ^f^	5
	4	8.1 ± 0.2 ^ef^	5	8.1 ± 0.4 ^ef^	5
	8	7.9 ± 0.5 ^cde^	5	7.9 ± 0.5 ^de^	5
	12	7.5 ± 0.6 ^cd^	4	7.4 ± 0.6 ^cd^	4
AGDC	0	8.4 ± 0.5 ^f^	5	8.5 ± 0.5 ^f^	5
	4	7.9 ± 0.3 ^def^	5	8.0 ± 0.4 ^ef^	5
	8	7.5 ± 0.6 ^cd^	4	7.3 ± 0.5 ^c^	4
	12	7.3 ± 0.5 ^bc^	4	6.7 ± 0.5 ^b^	3

OA—overall acceptability; MS—marketability score; C—uncoated control; AGSC—Aloe gel spray-coated; AGDP—Aloe gel dip-coated; different alphabets within the same column indicate significant difference at *p* ≤ 0.05 according to Tukey HSD test; data are means ± SD (*n* = 3).

## Supplementary Materials

The following are available online at https://www.mdpi.com/2304-8158/9/5/653/s1, Figure S1: Digital photographs showing effect of packaging on radish (RaS) and roselle (HbS) microgreens on 0 day and 8th day of storage, Figure S2: Digital photographs showing effect of edible coating techniques on radish (RaS) and roselle (HbS) microgreens on 0 day and 12th day of storage. Table S1: Effect of packaging on the marketability of radish and roselle microgreens during storage at 5 °C, Table S2: Effect of edible coating treatment on the marketability of radish and roselle microgreens during storage at 5 °C.
