# Supplementary material for: Effect of Packaging and Coating Technique on Postharvest Quality and Shelf Life of Raphanus sativus L. and Hibiscus sabdariffa L. Microgreens
Source: Foods. 2020 May 19;9(5):653. doi: 10.3390/foods9050653 (PMC7278862; doi:10.3390/foods9050653)
Supplement: Supplementary file 1 [file foods-09-00653-s001.pdf]

Supplementary materials

Table S1. Effect of packaging on the marketability of radish and roselle microgreens during storage at 5 °C.

Packaging	Storage period (Days)	% wilting	% leaf discoloration	% loss of OA	% loss of saleability	MS
			Radish microgreens			
PET- CS	0	0.00	0.00	7.8	1.6	5
	4	14.2	11.0	13.3	12.7	4
	8	35.2	15.1	22.2	24.6	3
LDPE - SSB	0	0.00	0.00	7.8	1.6	5
	4	18.8	12.0	16.7	15.7	4
	8	37.4	17.3	25.6	27.0	3
			Roselle microgreens			
PET- CS	0	0.00	0.00	6.7	1.3	5
	4	17.6	13.9	15.6	15.7	4
	8	37.4	17.6	26.7	27.3	3
LDPE - SSB	0	0.00	0.00	6.7	1.3	5
	4	19.9	16.0	17.8	17.9	4
	8	38.7	22.5	31.1	30.7	2

PET- CS: PET clamshell containers; LDPE – SSB: LDPE self-seal bags; OA: Overall acceptability; MS: Marketability score.

Figure S1. Digital photographs showing effect of packaging on radish (RaS) and roselle (HbS) microgreens on 0 day and 8th day of storage

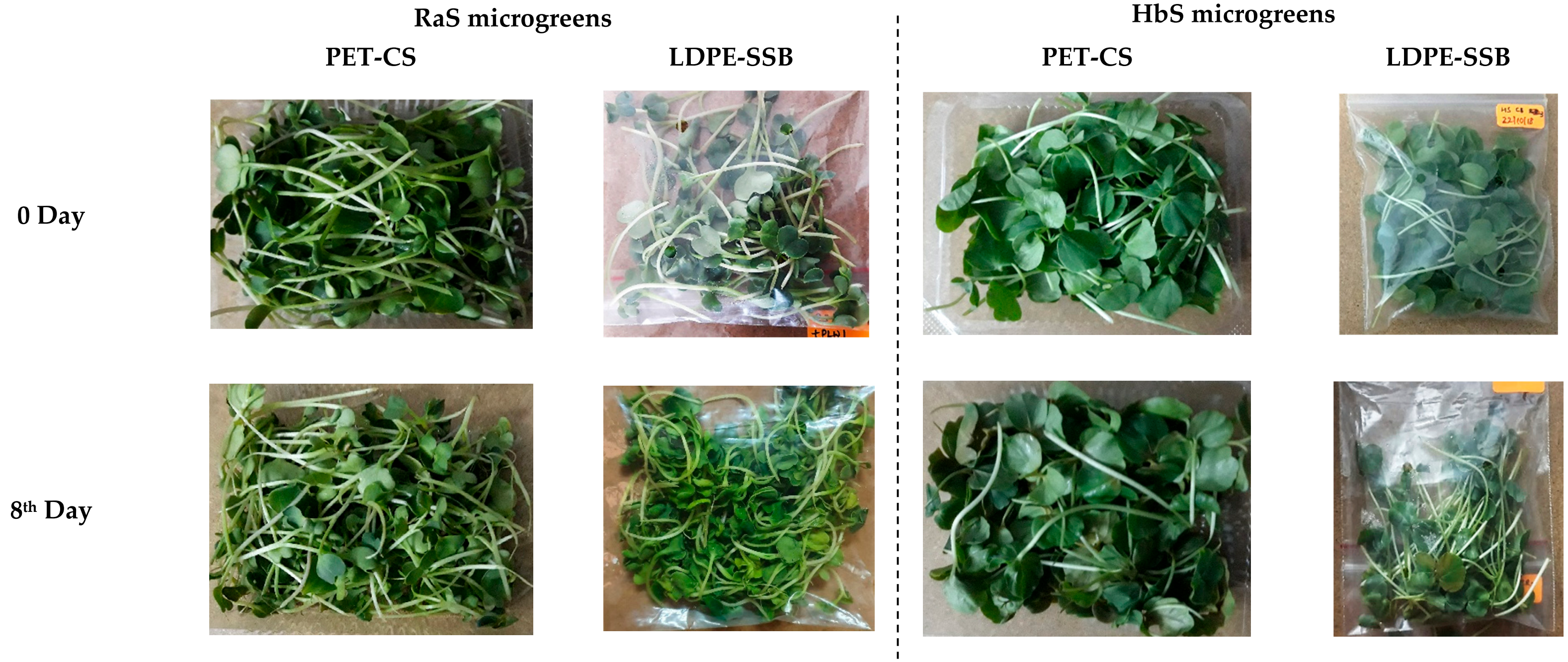

PET-CS: PET clamshell container; LDPE-SSB: LDPE self-seal bag

Table S3. Effect of edible coating treatment on the marketability of radish and roselle microgreens during storage at 5 °C.

**Packaging**	**Storage period (Days)**	**% wilting**	**% leaf discoloration**	**% loss of OA**	**% loss of saleability**	**MS**
				Radish microgreens		
C	0	0.0	0.0	7.8	1.6	5
	4	14.2	11.0	13.3	12.7	4
	8	35.2	15.1	22.2	24.6	3
	12	48.4	33.1	28.9	38.6	2
AGSC	0	0.0	0.0	6.7	1.3	5
	4	4.8	0.0	10.6	4.0	5
	8	12.8	2.3	12.2	8.5	5
	12	23.4	7.7	16.7	15.8	4
AGDC	0	0.0	0.0	7.1	1.4	5
	4	5.5	1.4	11.8	5.1	5
	8	16.4	5.1	16.4	11.9	4
	12	25.6	11.6	19.1	18.7	4
				Roselle microgreens		
C	0	0.0	0.0	6.7	1.3	5
	4	17.6	13.9	15.6	15.7	4
	8	37.4	17.6	26.7	27.3	3
	12	55.1	46.8	35.6	47.9	1
AGSC	0	0.0	0.0	5.6	1.1	5
	4	4.4	1.8	10.0	4.5	5
	8	10.2	7.8	12.8	9.8	5
	12	15.2	16.1	17.8	16.1	4
AGDC	0	0.0	0.0	5.6	1.1	5
	4	5.1	3.1	11.1	5.5	5
	8	12.7	15.8	18.9	15.2	4
	12	24.0	30.5	25.6	26.9	3

C – Uncoated control; AGSC – Aloe gel spray coating; AGDP – Aloe gel dip coating; OA – Overall acceptability; MS – marketability score.

Figure S2. Digital photographs showing effect of edible coating techniques on radish (RaS) and roselle (HbS) microgreens on 0 day and 12th day of storage.

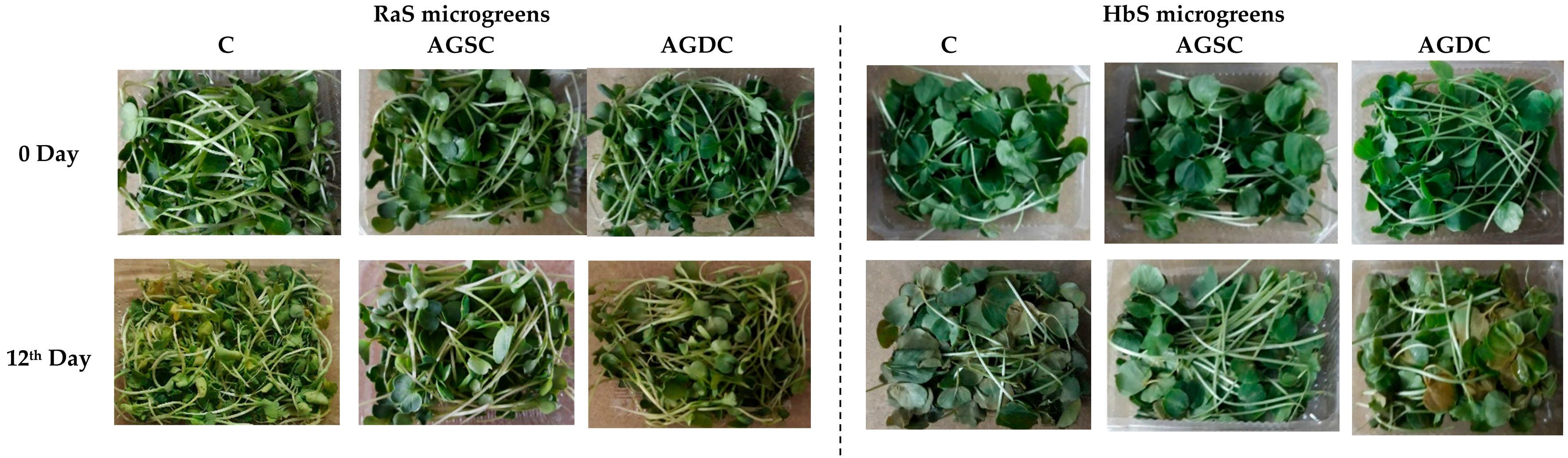

C: uncoated; AGSC: Aloe gel spray coating; AGDC: Aloe gel dip coating.
